# Traumatic neuroma of the medial antebrachial cutaneous nerve treated by targeted muscle reinnervation using the epitrochleoanconeus muscle

**DOI:** 10.1002/ccr3.9538

**Published:** 2024-11-24

**Authors:** Mark P. van Opijnen, Michel Wesstein, Godard C. W. de Ruiter

**Affiliations:** ^1^ Department of Neurosurgery Haaglanden Medical Center The Hague The Netherlands; ^2^ Department of Clinical Neurophysiology Haaglanden Medical Center The Hague The Netherlands

**Keywords:** anconeus epitrochlearis muscle, medial antebrachial cutaneous nerve, neuropathic pain, traumatic neuroma

## Abstract

This case shows the feasibility of targeted muscle reinnervation (TMR) in a patient with a traumatic neuroma of the medial antebrachial cutaneous nerve (MABCN). TMR was performed by connecting the proximal stump of the MABCN to the branch innervating the accessory epitrochleoanconeus muscle. Postoperatively, the patient reported significantly less pain.

## Graphical abstract

Intra‐operative image showing the technique of TMR for the MABCN to EAN.

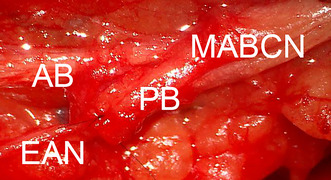

## 1 INTRODUCTION

Traumatic neuroma can develop after nerve injury if regenerating axons from the proximal nerve end do not reach the distal nerve stump. These neuromas can be extremely painful and may have a large impact on the quality of life.^1^, ^2^ There are several surgical options, depending on the availability of the distal nerve stump.^3^ If the nerve is unreconstructable because the distal nerve is not available or if the nerve gap or interval is too long, targeted muscle reinnervation (TMR) may be an option. This technique has been proved to be successful, both in the treatment of neuromas in amputees as well as for the treatment of single neuromas in nonamputees.^4^, ^5^, ^6^, ^7^ The principle of TMR is to direct the regenerating axons into another distal nerve target to prevent neuroma formation once the neuroma is resected.^7^ Several distal muscle targets have been described for various nerves,^8^, ^9^ but to the best of our knowledge no distal target has been suggested for the medial antebrachial cutaneous nerve (MABCN) in nonamputees. Several distal targets have been suggested in upper extremity amputation, including brachialis, brachioradialis and flexor digitorum profundus,^10^ but the disadvantage of using these targets in nonamputee single neuroma obviously is muscle weakness.

In this case report, we describe the potential use of the epitrochleoanconeus or anconeus epitrochlearis muscle as a target for TMR in the treatment of MABCN neuroma. The epitrochleoanconeus muscle (EAM) is a small, accessory muscle and an anatomical variant present in approximately 4%–34% of healthy individuals.^11^, ^12^ It spans from the medial epicondyle of the humerus to the olecranon and is innervated by the ulnar nerve (UN) running parallel to the UN over a relatively long trajectory.^13^ The role of the EAM in the development of cubital tunnel syndrome (CuTS) is double‐sided. The muscle may protect the UN, as suggested by Wilson et al.,^14^ but when it becomes bulky (hypertrophic), as in the case of strenuous exercise, it may lead to compression of the UN.^11^, ^15^ The present case shows the feasibility of connecting the proximal stump of the MABCN to the nerve branch innervating the EAM, the potential of preventing reformation of a neuroma, and the effect of denervation of the muscle on the underlying UN.

## 2 CASE PRESENTATION

### 2.1 Clinical history and course

**FIGURE 1:**
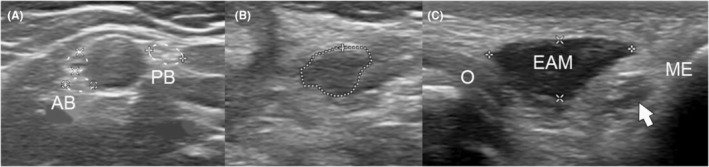
Preoperative ultrasound images. (A) Transverse image showing the anterior branches (AB) and posterior branch (PB) of the right medial antebrachial cutaneous nerve proximal to the lesion of the PB. (B) Transverse image showing the distal thickening (encircled) of the PB. (C) Transverse image showing the epitrochleoanconeus muscle (EAM) between the olecranon (O) and medial epicondyle (ME) with white arrow pointing at the underlying UN.

A man in his late 30s was referred to the senior author with two separate clinical problems: (1) cubital tunnel syndrome and (2) neuroma of the MABCN after a stab wound injury of his right arm more than 15 years ago. As for the first problem: he had symptoms of tingling in digits four and five for 1 year, with subjectively also decreased strength of his right hand. These symptoms were different from the decreased sensation in thumb, index finger, and middle finger, which he had since the stab wound injury and reconstruction of the median nerve in the acute phase elsewhere. On neurologic examination, there was decreased sensibility in the median nerve distribution of his right hand. He had no muscle weakness of his right hand. As for the second problem: over the years he had developed severe pain symptoms on the anteromedial side of his right arm, just above the elbow. The pain was constant, but increased with cold and pressure to nine points on the Visual Analogue Scale (VAS) despite the use of Pregabalin. Neurologic examination revealed a positive Tinel sign with radiating pain towards the medial side of the forearm in the sensory area of the MABCN. An ultrasound was performed which showed a neuroma of the posterior branch (PB) of the MABCN (with intact anterior branches, Figure 1A,B), 4 cm proximal to the cubital fossa. The UN was slightly thickened (cross‐sectional area [CSA] 9.6 mm^2^) and a bulky EAM was found (Figure 1C). Nerve conduction studies (NCS) showed a normal conduction velocity of the UN over the elbow.

Different surgical options were discussed with the patient (including decompression of the UN and resection of the neuroma with transposition of the proximal stump into triceps muscle) during multiple visits to the outpatient clinic. Because his symptoms of CuTS had improved over time, the option of TMR to the EAM was eventually chosen. Informed consent for the surgery was obtained and also for eventual case description.

### 2.2 Treatment

**FIGURE 2:**
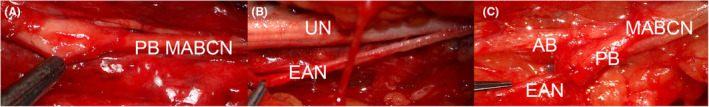
Intraoperative pictures of the targeted muscle reinnervation of the medial antebrachial cutaneous nerve (MABCN) to the branch to the epitrochleoanconeus muscle (EAM) in the right upper arm (left is distal, right is proximal). (A) The posterior branch (PB) of the MABCN with forceps pointing at the neuroma. (B) The branch to the EAM which runs parallel to the ulnar nerve (UN) proximal to the cubital tunnel. (C) The branch to the EAM that has been transposed and coaptated to the proximal end of the PB of the MABCN. As shown in Figure 1, the anterior branches (AB) of the MABCN were intact.

Intraoperatively, the MABCN was identified and thickening of the PB was seen at the site of the scar of the stab wound (Figure 2A). The UN was dissected in the upper arm in a distal direction towards the sulcus. The EAM was identified, as well as the nerve branch innervating the EAM. Bipolar stimulation of this branch (Neurosign bipolar probe) resulted in contraction of the EAM. The nerve branch was dissected proximally along the course of the UN (Figure 2B) to obtain sufficient length for the TMR procedure. After resection of the suspected neuroma of the MABCN, the branch to the EAM was transected 4–5 cm proximal to the innervation of the muscle and the end was transferred to the proximal end of the PB of the MABCN. The nerve ends were coaptated under the microscope (Zeiss OPMI Pentero 900) with two Ethilon 10–0 sutures. There was a good size‐match between the nerve to the EAM and the MABCN (Figure 2C).

### 2.3 Outcome and follow‐up

**FIGURE 3:**
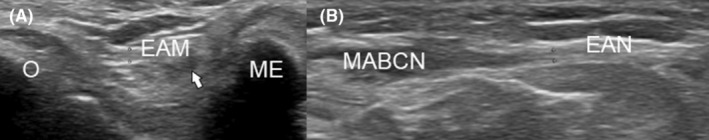
Postoperative ultrasound images. (A) Transverse image, obtained 1 year after the targeted muscle reinnervation. Clearly visible is the atrophy of the epitrochleoanconeus muscle (EAM). Thickness is shown by two white dots. The EAM runs between the olecranon (O) and medial epicondyle (ME). The ulnar nerve (white arrow) lies just lateral to the medial epicondyle under the EAM. (B) Longitudinal image, obtained 2 years after the targeted muscle reinnervation. On the left is the proximal stump of the medial antebrachial cutaneous nerve (MABCN), on the right is the branch to the EAM and in between the coaptation.

Postoperatively, the patient showed a good recovery and reported significantly less pain on the medial side of his right upper arm. Pathological analysis confirmed the diagnosis of a traumatic neuroma. One year after the TMR, the patient reported a good effect on the pain symptoms: he had no more pain in his right upper arm (VAS 0) and no symptoms of CuTS. Ultrasound and NCS were again performed. Ultrasound showed no thickening of the MABCN at the coaptation site to the nerve to the EAM, together with atrophy of the EAM (Figure 3A), and CSA of the UN was decreased compared to preoperatively (7.7mm^2^). NCS showed normal conduction velocity of the UN at the elbow. Two years after the TMR, another ultrasound was performed, still showing an intact coaptation (Figure 3B).

## 3 DISCUSSION

In this article, we presented the case of a traumatic neuroma of the MABCN nerve treated by TMR using the nerve branch to the EAM. This procedure resulted in successful pain relief, and denervation of the EAM did not lead to symptoms of CuTS.

To the best of our best knowledge, this is the first case report describing TMR for the treatment of a traumatic neuroma of the MABCN. Most frequently performed procedure for the treatment of MABCN neuroma is to transpose the proximal nerve end into the underlying triceps muscle. Previous studies have shown good results for this technique^16^ or did not specify the target muscle after recommending the implantation of the dissected end of the MABCN into a muscle.^17^, ^18^

Because the most frequent cause for MABCN neuroma is iatrogenic injury due to CuTS surgery we acknowledge that the potential for application of TMR to the nerve branch to the EAM is probably limited, because the EAM, if present, in these cases will have been transected or completely resected. Nevertheless, this case report shows that this technique can lead to successful recovery of pain symptoms. Moreover, our case also shows that denervation of the EAM did not lead to recurrence CuTS. It is difficult to say if the denervation contributed to the decrease in CSA of the UN, because the patient's symptoms of CuTS had already started to improve before the surgery. Selective denervation of the EAM could be an interesting treatment for patients with a bulky EAM, although more research is needed to further investigate this.

The present case illustrates the importance of noticing anatomical variations and how to take advantage of it once identified. Our case confirmed the previously described anatomical relation between the innervation of the EAM and the UN, which run together over a relatively long trajectory.^13^ According to our experience, sufficient length can be obtained to transfer the branch medially. Second, no increase in the cross‐sectional area (CSA) was found on postoperative sonographic analysis as well as normal nerve conduction. Finally, coaptation of the MABCN to the branch to the anconeus epitrochlearis muscle creates a good size‐match without new neuroma forming.

## 4 CONCLUSION

This case report shows a successful TMR in a patient with a traumatic neuroma of the MABCN 20 years after a stab wound. The anatomic variant EAM was used for the coaptation with the proximal end of the MABCN. Postoperatively, the patient reported a good effect on the pain symptoms in his right hand, and sonographic and nerve conduction analyses showed no new neuroma or ulnar neuropathy, in the presence of an atrophic EAM. Therefore, TMR can be a reasonable technique for the treatment of a traumatic neuroma of the MABCN in the presence of an EAM.

## AUTHOR CONTRIBUTIONS

**Mark P. van Opijnen:** Conceptualization; writing – original draft. **Michel Wesstein:** Data curation; visualization; writing – review and editing. **Godard C. W. de Ruiter:** Conceptualization; data curation; supervision; writing – review and editing.

## FUNDING INFORMATION

This study was funded by a grant from the Research Fund of Haaglanden Medisch Centrum. No grant number applicable.

## CONFLICT OF INTEREST STATEMENT

None of the authors declare a conflict of interest.

## CONSENT

Written informed consent was obtained from the patient to publish this report in accordance with the journal's patient consent policy.

## Data Availability

The data supporting the findings of this research are available upon reasonable request from the corresponding author.
